# Increasing Fluoroquinolone Resistance in *Campylobacter jejuni,* Pennsylvania, USA,1982–20011^1^

**DOI:** 10.3201/eid0812.020115

**Published:** 2002-12

**Authors:** Irving Nachamkin, Huong Ung, Ming Li

**Affiliations:** * University of Pennsylvania School of Medicine, Philadelphia, Pennsylvania, USA

**Keywords:** Campylobacter, fluoroquinolones, antimicrobial resistance, bacterial gastroenteritis

## Abstract

Fluoroquinolone-resistant *Campylobacter jejuni* has been observed worldwide and is now being seen in the United States. Among patients in our health-care system in Pennsylvania, fluoroquinolone-resistant *C. jejuni* were not observed from 1982 to 1992; however, resistance increased to 40.5% in 2001. Resistance to erythromycin remains at a low level (<5%).

*Campylobacter jejuni* is the most common cause of bacterial gastroenteritis in the United States, where an estimated 2.5 million cases occur each year (1). *Campylobacter* enteritis is primarily a foodborne illness; poultry is the major source for human infection (1). Most campylobacter infections need not be treated with antimicrobial agents; however, fluoroquinolones have been commonly used to treat serious *Campylobacter* infections and are also used as empiric therapy for travelers’ diarrhea (2).

Fluoroquinolone-resistant *C. jejuni* was recognized during the late 1980s in Europe, where researchers suggested that such resistance was due, in part, to acquisition of fluoroquinolone-resistant strains from animal sources (3). Smith and colleagues recently reported fluoroquinolone-resistant *C. jejuni* in Minnesota and found that, from 1992 to 1998, fluoroquinolone resistance increased from 1.3% to 10.2% (4). Recent data from the National Antimicrobial Resistance Monitoring System (NARMS) show that 14.2% of isolates submitted to the Centers for Disease Control and Prevention in 2000 were fluoroquinolone resistant (5). We have examined fluoroquinolone resistance and erythromycin resistance in *C. jejuni* isolated from patients seen at our institution since 1982. Previously we reported that fluoroquinolone resistance was not observed in isolates from 1982 to 1992 (6). In contrast to limited national data, we have observed a dramatic increase in fluoroquinolone resistance in *C. jejuni* since the mid-1990s.

## The Study

 The population we tested included patients treated by physicians within the University of Pennsylvania Health System, which encompasses several Philadelphia-area hospitals. Most isolates were from outpatients seen at the Hospital of the University of Pennsylvania or the Presbyterian Medical Center; both serve the University of Pennsylvania community and populations living in west Philadelphia. Stool samples were collected as part of the routine evaluation of patients with diarrheal illness and sent in Cary-Blair transport medium to the Clinical Microbiology Laboratory at the Hospital of the University of Pennsylvania for processing. *Campylobacter* organisms were isolated and identified to species by using published methods (7). Only *C. jejuni* subsp. *jejuni* were included in this study. Each isolate tested represents a single patient.

 From 1995 through 2001, 404 patient isolates were obtained from routine stool cultures; 297 (73.5%) were available for susceptibility testing. The ratio of males to females was 1.15:1. The age distribution was nearly identical for both sexes (males: median 33 yrs, mean 35 yrs [range 1–86 yrs]; females: medium 33 yrs; mean 36 yrs [range 8–95 yrs]). Isolates were stored at –70°C and subcultured at least once before testing. Susceptibility to ciprofloxacin and erythromycin was determined with the E-test (AB Biodisk, Solna, Sweden) method. Organisms were tested on Mueller-Hinton blood agar medium and incubated at 37°C in microaerobic conditions. The breakpoints used for resistance were ≥4 μg/mL for ciprofloxacin and ≥8 μg/mL for erythromycin (5). Flagellin gene typing (Fla typing) was performed by using modified consensus primers, described by Wassenaar and Newell (8), and digested with *DdeI* as previously described (9).

**Figure 1:**
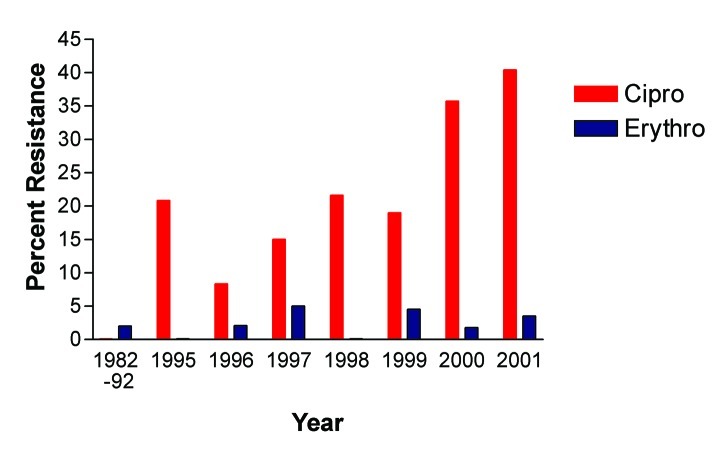
Trends in erythromycin and ciprofloxacin resistance in *Campylobacter jejuni,* Philadelphia*,* 1982–2001. Number of isolates tested: 1982–92 (n=142), 1995 (n=24), 1996 (n=48), 1997 (n=61), 1998 (n=37), 1999 (n=22), 2000 (n=48), and 2001 (n=47).

**Figure 2:**
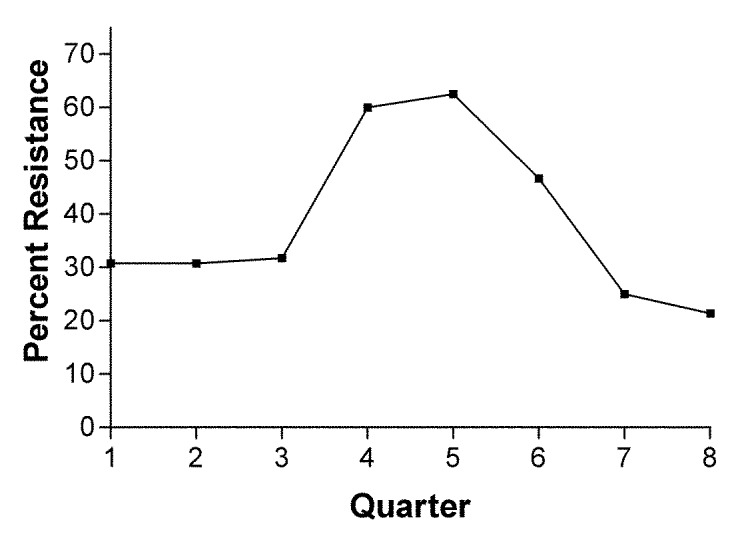
Fluoroquinolone-resistant *Campylobacter,* by quarter, 2000–2001. Number of isolates tested for each quarter: Q1: 13, Q2: 13, Q3: 22, Q4: 10, Q5: 16, Q6: 15, Q7: 12, Q8: 14.

 As reported previously, fluoroquinolone-resistant *C. jejuni* were not detected among 142 patient isolates tested from 1982 to 1992 at our institution (6). Erythromycin resistance was 2.0% overall from 1982 to 1992. Two hundred and ninety-seven patient isolates were tested between 1995 and 2001 for susceptibility to ciprofloxacin and erythromycin. Resistance rates ranged from as low as 8.3% in 1996 to 40.5% in 2001 (Figure 1). In contrast, erythromycin resistance fluctuated between 0% and 5% during the same period; in 2001 erythromycin resistance was 3.5%. When all isolates tested during the study period are considered, 28.9% of isolates were resistant in the first calendar year quarter, 19.7% in the second quarter, 20% in the third quarter, and 19.2% in the fourth quarter. However, resistance isolates were more frequent beginning in October 2000 and extending through April 2001 (Figure 2).

**Figure 3:**
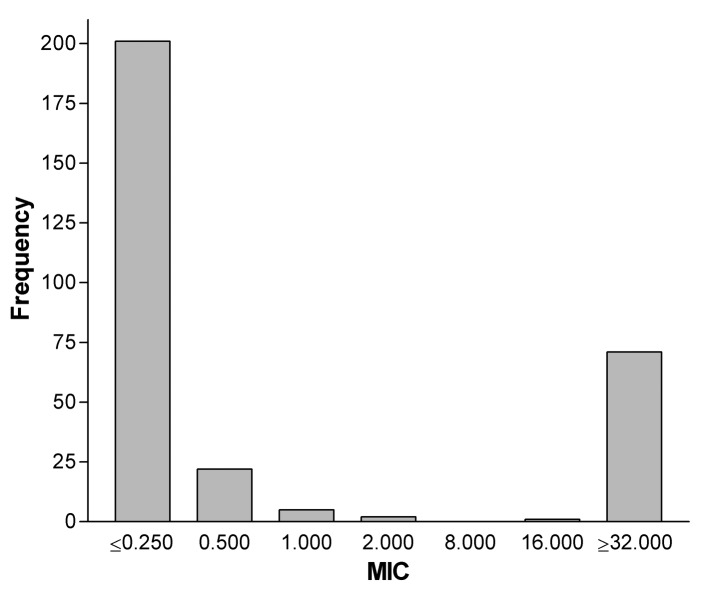
Distribution of ciprofloxacin MICs in *Campylobacter jejuni,* 1995–2001.

Figure 3 shows the ciprofloxacin MIC distribution of isolates from 1995 to 2001. A clear bimodal distribution of MICs exists, with 96% of susceptible isolates with MICs ≤0.5 μg/mL; except for one isolate, all resistant isolates had MICs ≥32 μg/mL.

 We used molecular typing by restriction fragment length polymorphism analysis of *Campylobacter* *flaA* to determine whether certain Fla types were associated with fluoroquinolone resistance. Twenty-nine different Fla types occurred among the population of isolates. For strains there were at least four isolates represented in the type (Fla types 1, 7, 9, 10, 13, 15, 16, 25, 33, 44, 48, 49, 53, 57, 80, 86). The proportion of resistant to susceptible isolates was no more than 0.25 and ranged from 0.07 to 0.25. None of the Fla types were specifically associated with fluoroquinolone resistance.

## Conclusions

 We have observed a dramatic increase in fluoroquinolone-resistant *C. jejuni* in patients treated within our health system from 1995 to 2001 with a resistance rate of 40.5% in 2001. In contrast, erythromycin-resistant *C. jejuni* has remained at a low rate (<5%) for nearly 20 years. Before 1992, fluoroquinolone-resistant *C. jejuni* had not been detected at our institution (6). Whether fluoroquinolone resistance emerged during 1993–1994 is unknown because isolates from that period were not available. Similarly, a survey of isolates from 19 U.S. counties in 1989 and 1990 did not find any fluoroquinolone-resistant *C. jejuni* (1). From 1997 through 2000, NARMS reported 13%, 13%, 18 %, and 14% fluoroquinolone-resistant *C. jejuni*, respectively (5). Erythromycin-resistant *C. jejuni* occurred in 8%, 3%, 2%, and 1% of isolates from 1997 to 2000 among isolates tested by NARMS; our data parallels these national data (5). The distribution of ciprofloxacin MICs among *C. jejuni* from our survey also parallels NARMS data between 1997 and 2000 (5). Fluoroquinolone-resistant isolates exhibited high-level resistance with MICs >32 μg/mL.

 The risk factors for acquiring fluoroquinolone-resistant *C. jejuni* in the United States have not been defined; however, foreign travel was identified by Smith and colleagues as an important risk factor (for 75% of fluoroquinolone-resistant *C. jejuni*) in Minnesota residents (4). Other, unidentified factors were important, however, since the rest of infections were domestically acquired. Use of a fluoroquinolone within the month before the collection of the stool sample was also identified as a potential risk factor (4). The increase in fluoroquinolone-resistant *C. jejuni* from 1996 through 1998 was temporally associated with the licensure of fluroquinolones for use in poultry in the United States (4). Several studies from European colleagues noted this temporal relationship between use of fluoroquinolones in animals and resistance among human isolates in the 1980s (3).

 The reasons for such a dramatic increase in fluoroquinolone-resistant *C. jejuni* in our population are unknown. We examined the connection between seasonality and isolation of fluoroquinolone-resistant *C. jejuni.* We did observe increasing rates of resistance for several quarters during the last 2-year survey period. Whether this increase is indicative of foreign travel patterns by our patients is unknown. Future studies should focus on identifying the factors for acquisition of fluoroquinolone-resistant *C. jejuni* as well as the clinical implications of infection with such strains. Some evidence suggests that infection with fluoroquinolone-resistant *C. jejuni* results in prolonged illness. The duration of diarrhea among patients treated with a fluoroquinolone in the Minnesota study was significantly longer if the patient had a fluoroquinolone-resistant infection (median 10 days) versus a fluoroquinolone-susceptible infection (median 7 days)(4). Based on national trends and our own local data, erythromycin continues to be the drug of choice for treating *Campylobacter* gastroenteritis.
